# Decreased Sperm Motility Retarded ICSI Fertilization Rate in Severe Oligozoospermia but Good-Quality Embryo Transfer Had Achieved the Prospective Clinical Outcomes

**DOI:** 10.1371/journal.pone.0163524

**Published:** 2016-09-23

**Authors:** Jufeng Zheng, Yongning Lu, Xianqin Qu, Peng Wang, Luiwen Zhao, Minzhi Gao, Huijuan Shi, Xingliang Jin

**Affiliations:** 1 Key Laboratory of Contraceptive Drugs and Devices, Shanghai Institute of Planned Parenthood Research, Shanghai, China; 2 Department of Reproductive Medicine, Renji Hospital, Shanghai Jiaotong University, Shanghai, China; 3 Shanghai Key Laboratory of Reproductive Medicine, Shanghai Jiaotong University Medical School, Shanghai, China; 4 Sydney Center for Regenerative and Developmental Medicine, Kolling Institute for Medical Research, Sydney Medical School, University of Sydney, St. Leonards, NSW, Australia; 5 Reproductive Health Center, the Second Affiliated Hospital of Wenzhou Medical University, Wenzhou, China; Universite Blaise Pascal, FRANCE

## Abstract

**Introduction:**

Spermatozoa motility is the critical parameter to affect the treatment outcomes during assisted reproductive technologies (ART), but its reproductive capability remains a little informed in condition of severe male factor infertility. This retrospective cohort study aimed to evaluate the effects of reduced sperm motility on the embryological and clinical outcomes in intra-cytoplasmic sperm injection (ICSI) treatment of severe oligozoospermia.

**Patients and Methods:**

966 cycles (812 couples) of severe oligozoospermia diagnosed by spermatozoa count ≤ 5 × 10^6^/mL and motile spermatozoa ≤ 2 × 10^6^/mL were divided into four groups in according to the number of motile spermatozoa in one ejaculate on the day of oocyte retrieval (Group B—E). The control (Group A) was 188 cycles of moderate oligozoospermia with spermatozoa count > 5 × 10^6^/mL and motile spermatozoa > 2 × 10^6^/mL. All female partners were younger than 35 years of age. Logistic regression analyzed embryological outcomes (the rates of fertilization, cleavage and good-quality embryo) and clinical outcomes (the rates of pregnancy, implantation, early miscarriage and live birth). Quality of embryo transfer (ET) was divided into three classes as continuous factor to test the effects of embryo quality on clinical outcomes.

**Results:**

The reduction in the number of motile sperm in four groups of severe oligozoospermia gave rise to comparable inability of the fertilization (p < 0.001) and a decreased rate of good-quality embryo at Day 3 (p < 0.001) by compared to the control. The cleavage rate of the derived zygotes was similar to the control. ET classes significantly affected the clinical outcomes (p < 0.001). Class I ET gave rise to similar rates of clinical outcomes between five groups, but Class II and Class III ET retarded the rates of pregnancy, implantation and live birth and this particularly occurred in Group C, D and E. The rate of early miscarriage was not comparably different between groups. Overall rates in all groups were 41.26% clinical pregnancy, 25.74% implantation and 36.32% live birth, which gave live birth to 252 girls and 252 boys.

**Conclusions:**

The reduction of motile spermatozoa in severe oligozoospermia decreased the rates of fertilization and good-quality embryo. Obtaining and transfer of good-quality embryos was the good prognostic to achieve prospective clinical outcomes regardless of the severity of oligozoospermia.

## Introduction

Near half of infertility is due to inability of males to achieving pregnancy in a fertile female [1, 2]. Many cases of male infertility are poorly understood and recent ability to diagnose these defects remains limited [3–5]. Clinically, the sperm parameters including motility, number and morphology are used to identify males with subfertility and infertility, which is commonly described as dominant parameters indicating the clinical outcomes of IVF and ICSI [6, 7]. But male patients are not regularly followed up and the prognosis of the male infertility is a little informed. Oligozoospermia is one of the most common causes of male factor infertility and refers to semen with a low concentration of sperm (≤ 20 × 10^6^/mL) [8–11]. The concentration less than 5 × 10^6^ /mL is recognized as severe condition, but the severity of this condition affecting the reproductive viability is not clearly defined. 10 million motile sperm is commonly suggested be a threshold of satisfying ICSI fertilization rates performed with ejaculated spermatozoa [12, 13]. In a number of situations direct medical or surgical intervention can improve the sperm concentration and even pregnancy rate; examples are use of FSH (follicle-stimulating hormone) in men with pituitary hypogonadism, antibiotics in case of infections, or surgical corrections of a hydrocele, varicocele, or vas deferens obstruction [14, 15]. Notably, the choice of treatment and management can be complex as the causes of the conditions are the fecundity of the female partner also to be considered. In many cases with oligozoospermia, IVF—ICSI is done and is often the best option, specifically if time is a factor or fertility problems coexist on the female side.

Recent advances in ICSI technique can treat severe infertile men with oligo-astheno-teratozoospermia, azoospermia, cryptozoospermia and necrospermia [16–18] to achieve fatherhood. However, in the case with extremely severe oligozoospermia, isolation and preparation of motile sperm for ICSI are difficult. Optional treatment of these cases is usually using testicular sperm extract (TESE) or donor sperm. The fact is that the parameter of TESE in the oligozoospermia patients without obstructive factor is obviously worse than the ejaculate semen, and immature TESE gametes may cause lower reproductive ability [19, 20] and the risks of potential imprinting defects [21–23]. Utilization of own gametes instead of donors to become a biological parent is always an aspiration for the couple. Given the fact that ICSI technique theoretically requires only one piece of motile sperm to allow infertile men to achieve fatherhood [16, 24], it is warranted to evaluate clinical outcomes of ICSI under extreme situations where only several pieces of motile sperm were found in one ejaculate in the severe male infertility. However, conclusive data about the indication of motile sperm count on ICSI treatment of such cases remains limited. This retrospective study analyzed the outcomes of ICSI treatment of severe oligozoospermia by deep classification of its severity based on the number of motile sperm in one ejaculate on the day of oocyte retrieval. Without consideration of the influences that might be resulted from female partners, this study supplied a significantly indicative support for the utilization of ICSI technique in the treatment of severe male factor infertility.

## Materials and Methods

### Ethics

The retrospective cohort analysis on embryological and clinical outcome after ICSI insemination was conducted in accordance with the research protocol approved by the Ethical Review Committee, Renji Hospital, Medical school of Shanghai Jiaotong University, People’s Republic of China. Patients undergoing ICSI agreed with the proceedings and signed the informed agreements elucidated in the Clinical Informed Consent for ART devised by Department of Reproductive Medicine, Renji Hospital. The signed agreements were recorded and saved together with patients’ documents.

### Patients and oligozoospermia grouping

Between January 2003 and December 2010, 3519 IVF cycles, 3462 ICSI cycles and 394 half IVF/ICSI cycles were performed in our center. The authors had had access to identifying information after data collection for this this (S1 File). 188 cycles (164 couples) from 830 ICSI cycles of mild and moderate male infertility were used for control (Group A) and 966 (812 couples) out of 1119 severe oligozoospermia were divided into four groups (Group B—E) in according to the number of motile spermatozoa from one ejaculate on the day of oocyte retrieval (Table 1). All female partners were younger than 35-year old without any factors of female infertility other than fallopian tubes damage or blockage.

**Table 1:** Characteristics of the couples and ICSI cycle number in five groups of oligozoospermia.

Group	Cycle	Total sperm (per mL)	Motile sperm (A+B+C) (per mL)	Male age (years old)	Female age (years old)	Infertility duration (years)
**A**	188	> 5 x 10^6^	> 2,000,000	32.35 ± 0.38	29.56 ± 0.22	4.53 ± 0.18
**B**	118	≤ 5 x 10^6^	1,000,001 ~ 2,000,000	31.41 ± 0.37	29.13 ± 0.27	4.20 ± 0.24
**C**	489	≤ 5 x 10^6^	100,001 ~ 1,000,000	31.48 ± 0.19*	28.88 ± 0.13*	4.07 ± 0.11*
**D**	203	≤ 5 x 10^6^	10,001 ~ 100,000	30.47 ± 0.27*	28.31 ± 0.19*	4.14 ± 0.20*
**E**	156	≤ 5 x 10^6^	≤ 10,000	31.11 ± 0.33*	28.83 ± 0.22*	3.96 ± 0.18*
**Total**	1154	-	-	31.38 ± 0.13	28.91 ± 0.09	4.15 ± 0.07

Oligozoospermia was divided into five groups in according to sperm count and number of motile sperm in ejaculated semen. Mean ± SEM of the couple’s age and infertility years were displayed.

*p < 0.05, compared with corresponding data Group A.

### Semen examination and classification

The sperm parameter was assessed in according to evaluation of the semen parameters and processing of human semen [25]. The number of motile sperm in Group B to E was manually counted with Makler count chamber [26] under invert microscope (NIKON, Japan) with a microscopic objective of × 20 (200 HP magnification) instead of conventional CASA method. Group B and C were defined in according to the number of motile sperm counted in one field. Group D was recognized when at least one piece, but less than ten of motile sperm was observable in ten fields. Because Group E was lower than 1 × 10^4^/mL and no motile sperm could be found in ten fields, the sample was rinsed with HEPES-modHTF medium and centrifuged to concentrate spermatozoa. The small pellet was re-suspended to count sperm with above methods. For those with dozens of sperm, total of the motile sperm in all fields was counted. The urine including sperm from retrograde ejaculation was centrifuged immediately to obtain sperm pellet that was then suspended in 1 mL HEPES-modHTF for parameter assessment.

### Andrological preparation and ICSI treatment

**Fig 1:**
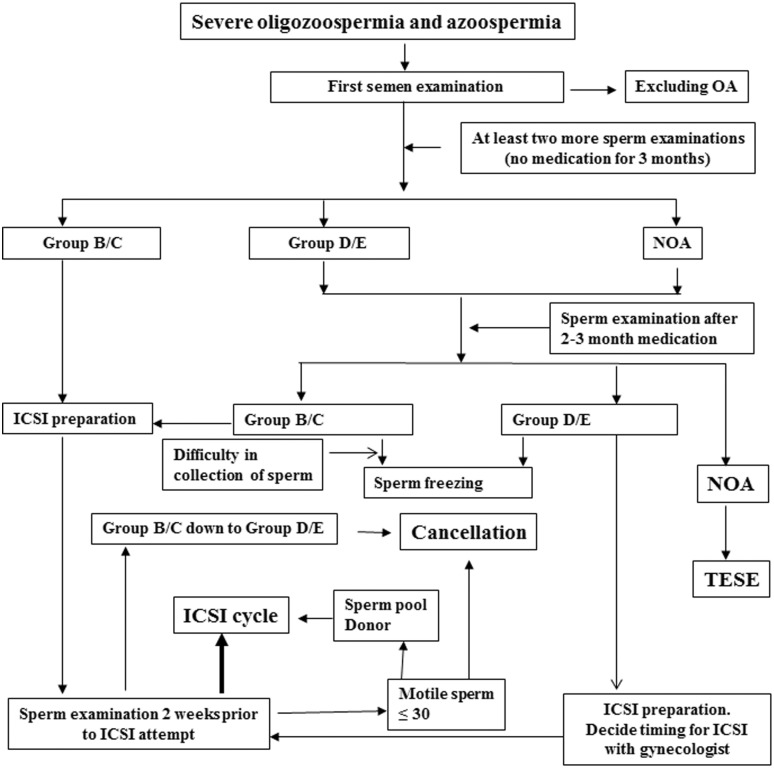
A proposal program of management of severe oligozoospermia and azoospermia. After differential diagnosis of obstructive azoospermia (OA), severe oligozoospermia was classified in according to the number of motile sperm from at least two ejaculates 4 weeks prior to ICSI attempt by andrologists. Proper medication might improve sperm motility. Cryopreservation was done for those having difficulty of collecting semen by masturbation and for Group D and E (motile sperm ≤ 1 × 10^5^). Semen had to be examined again 2 weeks prior to ICSI attempt. ICSI might be suspended if motile sperm in Group B and C (motile sperm > 1 × 10^5^) was down to the level of Group D and E, and these cases required further examination to determine next managements. Optional ICSI with patients’own pooled sperm or donor sperm, and cancellation of ICSI attempt was mainly recommended in the cases with extremely low number of motile sperm (≤ 30 in total). Non-obstructive azoospermia (NOA) could be treated with TESE if motile sperm was available.

Proposed diagnosis and management of severe oligozoospermia were routinely performed in our center (Fig 1). This was well organized and required good teamwork from andrologists, embryologists and clinicians. Differential diagnosis to exclude obstructive infertile factor, accurate classification of severe oligozoospermia at several stages, suitable medication to improve sperm motility, sperm cryopreservation, determining the timing entering ICSI cycle, management of excess oocytes and cancellation of ICSI attempt were key issues. Cancellation of ICSI attempt might be optional in according to patients’ agreement. All male partners accepted karyotype examination and most of them were analyzed for azoospermia factor (AZF) microdeletion as well.

The semen preparation and ICSI procedures were described [18]. In Group D and E, the one with better sperm quality from two or more samples was used for the ICSI treatment but all motile sperm might be used in Group E. Group E was suggested to collect semen at least twice in a row with skipped semen assessment to avoid wasting sperm, especially for those total mobile sperm count ≤ 30 pieces indicated by the latest assessment. The pellet was resuspended in 0.2 mL medium to be easily picked up for ICSI. The first step was to collect motile sperm and then determined the number of oocytes to be microinjected. The excess oocytes might be microinjected with sperm from their own frozen pool or with donor sperm; otherwise they were cryopreserved.

### Embryo transfer and clinical outcomes

The good-quality embryo at Day 3 preferred for ET, but the relative lower quality embryo might be also transferred as there were no enough standard good-quality embryos chosen from and/or the couple requested to increase successful opportunity. The quality of ET was classified into three classes: Class I ET was performed with standard good-quality embryo(s) only; Class II with at least one good-quality embryo and the rest lower quality; Class III with all lower quality embryo(s). Urinary HCG levels were measured two weeks after transfer to diagnose pregnancy. The clinical outcomes were assessed as described [18], and implantation rate was also reviewed.

### Statistical Analysis

The rates of embryological and clinical outcomes were evaluated using binary logistic regression analysis with SPSS for Window (Version 21.0, SPSS Inc., Chicago, IL, USA). The rates depending to the given landmark were set as the dichotomous dependent variable, and the oligozoospermia groups, ET classes and male age as covariates. The oligozoospermia groups were also set as a categorical covariate to contrast the differences between the oligozoospermia groups and their interaction with other covariates. The number of MII oocytes, years of age and infertility duration and the number of ET were analyzed by Univariate analysis of variance. Difference between individual oligozoospermia group was assessed by the least significance test where indicated.

## Results

Characteristics of the patients in five oligozoospermia groups were summarized (Table 1). The ages and infertility years in Group A were greater than Group C, D and E (p < 0.05), but no difference between Group B, C, D and E. The cycle frequency was relatively even distributed beyond the means of couples’ age with a range of female age from 21 to 34, and male age from 22 to 59; and no particular age group displayed the alterative cycle frequency.

Total 11584 MII oocytes were microinjected with patients’ sperm (Table 2). 27 cycles in Group E having excess sibling MII oocytes were microinjected with donor sperm due to lack of enough number of patients’ motile sperm. The results had been merged to our previous publication [18] and were not included in this study. A significant reduction in the rate of successful fertilization to form PN2 embryos was found in four groups of severe oligozoospermia compared to the control (p < 0.001). The severity of this fertilization inability tended to be associated with the reducing number of the motile spermatozoa. The fertilized eggs from these four groups of severe oligozoospermia had similar ability to perform cell cleavage by compared to the control (p > 0.05). The rate of observed good-quality embryo at Day 3 in four groups of severe oligozoospermia was fluctuant, but each was lower than the control (p < 0.001). The embryological outcomes were not affected by male age (p > 0.05).

**Table 2:** Embryological outcomes of ICSI treatment of oligozoospermia.

Group	MII		Fertilization rate		Cleavage rate		Good-quality rate	
	Mean	N	Mean	N	Mean	N	Mean	N
**A**	9.08	1707	0.8219	1403	0.9722	1364	0.5674	774
**B**	10.08	1190	0.7916*	942	0.9766	920	0.4793**	441
**C**	10.34	5058	0.7849*	3970	0.9751	3871	0.5045**	1953
**D**	10.95	2222	0.7538*	1675	0.9594	1607	0.5302**	852
**E**	9.02***	1407	0.7541*	1061	0.9698	1029	0.5053**	520
**Total**	10.04	11584	0.7813	9051	0.9713	8791	0.5164	4540

Displayed were the mean and total number of microinjected MII oocytes, the mean rates and total number of fertilized 2PN oocytes, cleavage embryos and good-quality embryos in five groups of oligozoospermia.

*p < 0.001,

**p < 0.001, compared to corresponding data in Group A (Control);

*** The presented number did not comprise the oocytes microinjected with donor sperm.

ET was performed with the highest quality embryos derived from couples’ own origin at Day 3 in 1093 cycles. Similar number of embryos was transferred in all groups of oligozoospermia (p > 0.05) (Table 3). 848 cycles were Class I ET. The proportion of Class I ET in four severe oligozoospermia groups was lower than control with significant reduction in Group E (p < 0.05), indicating a reduced opportunity to choose good-quality embryo for transfer. The rests were 160 cycles of ET Class II and 85 cycles of ET Class III (Table 3). Without consideration of ET classes, transferred embryos had similar ability to be pregnant and implanted in four groups of severe oligozoospermia and control and did not affect early miscarriage rate. As a consequence, the rate of live birth was not significantly different between five groups of oligozoospermia. The clinical outcomes were not statistically correlated with male age.

**Table 3:** The association of embryo transfer quality with clinical outcomes of ICSI treatment of oligozoospermia.

Group	ET class	ET class proportion	ET			Clinic pregnancy		Implantation		Early miscarriage		Live birth	
			Cycle	Mean	N	Mean	N	Mean	N	Mean	N	Mean	N
**A**	**I**	0.8315	148	2.01	298	0.4054	60	.2651	79	.0833	5	.3581	53
	**II**	0.1011	18	2.22	42	0.4444	8	.2143	9	.0000	0	.4444	8
	**III**	0.0674	12	2.17	24	0.2500	3	.1667	4	.0000	0	.2500	3
	**Total**	1.0000	178	2.04	364	0.3989	71	.2527	92	.0704	5	.3596	64
**B**	**I**	0.7679	86	2.01	173	0.4535	39	.2948	51	.1538	6	.3721	32
	**II**	0.1429	16	2.27	39	0.3750	6	.2308	9	.0000	0	.3750	6
	**III**	0.0892	10	2.18	19	0.1000	1	.0526	1	.0000	0	.1000	1
	**Total**	1.0000	112	2.06	231	0.4107	46	.2641	61	.1304	6	.3482	39
**C**	**I**	0.7689	356	2.10	747	0.4803	171	.3106	232	.0643	11	.4326	154
	**II**	0.1490	69	2.01	161	0.2609	18	.1491	24	.0556	1	.2319	16
	**III**	0.0821	38	2.42	70	0.2105	8	.1286	9	.0000	0	.2105	8
	**Total**	1.0000	463	2.11	978	0.4255	197	.2710	265	.0609	12	.3844	178
**D**	**I**	0.7959	156	2.12	328	0.4551	71	.2713	89	.0845	6	.3846	60
	**II**	0.1327	26	2.15	60	0.2692	7	.1667	10	.0000	0	.2692	7
	**III**	0.0714	14	2.43	30	0.1429	2	.1000	3	.500	1	.0714	1
	**Total**	1.0000	196	2.14	418	0.4082	80	.2440	102	.0875	7	.3469	68
**E**	**I**	0.7083*	102	2.02	206	0.4314	44	.2621	54	.0909	4	.3824	39
	**II**	0.2153	31	2.11	75	0.3548	11	.1867	14	.3636	4	.2258	7
	**III**	0.0764	11	2.53	20	0.1818	2	.1000	2	.0000	0	.1818	2
	**Total**	1.0000	144	2.09	301	0.3958	57	.2326	70	.1404	8	.3333	48
**Total**	**I**	0.7758	848	2.07	1752	0.4540	385	.2882	505	.0831	32	.3986	338
	**II**	0.1464	160	2.10	377	0.3125	50	.1751	66	.1000	5	.2750	44
	**III**	0.0778	85	2.38	163	0.1882	16	.1166	19	.0625	1	.1765	15
	**Total**	1.0000	1093	2.10	2292	0.4126	451	.2574	590	.0843	38	.3632	397

Displayed was the classification of the embryo transfer and the clinical outcomes.

* p < 0.05, proportion of ET classes, compared to the corresponding data in Group A. The mean, total cycle and total number of ET, the mean rate and number of pregnancy, implantation and live birth were displayed, based on the total cycles of three ET classes in five groups of oligozoospermia. The rate of miscarriage was the mean of the proportion of miscarriage cycle in all of the pregnant cycles. The relevant p values were displayed in the text.

Furthermore, ET classes were used as continuous factor for statistical analysis to test our hypothesis that transfer with good-quality embryo would reach prospective clinical outcomes in severe oligozoospermia (Table 3). The results showed that Class I ET achieved the similar clinical outcomes between five groups of oligozoospermia. Class II and Class III ET caused the comparable reduction rates of pregnancy (p < 0.001), implantation (p < 0.001) and live birth (p < 0.01). Importantly, the effects of ET classes on clinical outcomes were not interactive to the groups of oligozoospermia (p > 0.05). The detailed outcomes after pregnancy was shown (Tables 3 and 4). There was no significant difference between baby genders. The excess embryos were cryopreserved and clinical outcomes from frozen embryo transfer (FET) were showed (S2 File).

**Table 4:** Detailed clinical achievement after pregnancy.

Group	Pregnancy cycle	Miscarriage			Live birth			Baby gender	
		Early	Mid-late	Ectopic pregnancy	withdraw	Single	Multiple	Male	Female
**A**	71	5	0	2	2	45	17	42	37
**B**	46	6	0	1	2	23	14	26	25
**C**	197	12	3	4	6	116	56*	113	116
**D**	80	7	3	2	0	52	16*	40	45
**E**	57	8	0	1	0	36	12	31	29
**Total**	451	38	6	10	10	272	115	252	252

The table was representative of achieved cycles of miscarriage and live-birth, and baby gender.

*All were doublet except one triplet.

Karyotype was examined in all cases and no abnormality was found. The number of cases with AZFc microdeletion was 3, 3 and 1 in Grade B, C and E, respectively. 4 cases with AZFc microdeletion from Grade A and B achieved pregnancy, and gave birth to 5 in 3 cases. In Grade E, excess oocytes were microinjected with donor sperm in 27 cycles. 2 cycles had insufficient number of motile sperm and some oocytes were cryopreserved. One of them was failed with testicular sperm extraction (TESE). 10 oocytes were donated from 28 retrieved oocytes in one cycle. There were two cycles to try to use patient pooled sperm. One was failed to recover, and another had three of five oocytes fertilized, and the rest sibling oocytes were microinjected with donor sperm. 2 cycles in Group A were successfully microinjected with patients’ pooled sperm because of difficulty of sperm collection by masturbation and one cycle gave rise to a twin.

## Discussion

This study demonstrated the reduced number of motile spermatozoa had declined the fertility ability and embryo quality during ICSI treatment of severe oligozoospermia. The crucial finding was that the prospective clinical outcomes could be achieved as long as well selected good-quality embryos were transferred, regardless of whatever severity of oligozoospermia. In contrast, transfer of lower quality embryos reduced the rates of pregnancy, implantation and live birth. This provided a well informed prognostic parameter for ART clinic. Very a few studies have addressed the influence of the varying number of motile spermatozoa in the outcomes of ICSI treatment of severe oligozoospermia, especially in the case of motile sperm less than 10^4^/mL. This study gave rise to a relatively conclusive insight into the objective of the retrospective analysis by deep classification of the severity of the disease in term of sperm motility.

Oligozoospermia is greatly due to testicular or spermatogenic failure [27]. Tendency to a gradual reduction of the fertilization rate associated with severity of oligozoospermia illustrated the spermatogenic function status in individual oligozoospermic patient. As a consequence, this deteriorated the quality of derived embryos and reduced the opportunity to choose good-quality embryo for transfer.

Overall analysis of clinical outcomes veiled the disadvantages of low quality ET, largely due to more than two embryos having been transferred with Class I ET in most cycles. Analysis from 245 cycles transferred with lower class ET unveiled the adverse clinical outcomes and this apparently occurred in Group C, D and E. The implantation rate is the important parameter to evaluate the ability of individual embryo to be implanted and was not associated with sperm motility in this study, suggesting relatively similar quality in individual good-quality embryo had been obtained in all groups so that it led to similar prospective rate of live birth. Miscarriage is thought to have multiple etiologies, including parental chromosomal outubeanomalies, maternal thrombophilic disorders, immune dysfunction and various endocrine disturbances [28]. No statistical difference in the rate of early miscarriage was found between groups, because young and healthy female partners limited age-associated first trimester spontaneous miscarriage [29, 30], and male age had no comparable effects on embryological and clinical outcomes.

To limit the cancellation due to azoospermia on the day of ICSI attempt, using patients’ frozen pooled sperm, TESE and sperm donor are optional. Cryopreservation of patient’s sperm is strongly recommended and beneficial [31]. In our center, this was mainly for the cases having difficulty of collecting by masturbation. We found: (1) as long as careful diagnosis before ICSI attempt and correct preparation of semen were done with our proposed procedures, sufficient number of motile sperm in Group A to D might be isolated for microinjection; (2) the frozen sperm were much harder to obtain a successful microinjection comparing to the freshly isolated sperm and this was particular in Group E; (3) for those with ≤ 30 motile sperm, there was a little possibility to have enough sperm left for cryopreservation after the sample had been used for assessment; (4) the failure to recover sperm from cryopreservation was also mentioned. Therefore an advanced cryopreservation for single sperm is worth creating. TESE demonstrates the good results in azoospermia [32] and severe necrozoospermia [33], but it was found difficultly to be managed, especially for Group E and its superiority was also questioned [31]. Only one case of azoospermia occurred on ICSI day instead of using frozen sperm and donor sperm.

Successful ICSI was greatly attributed to excellent management of the patients prior to ICSI attempt and appropriate manipulation of all procedures by experienced andrologists, embryologists and gynecologists (Fig 1). Arrangement of proper timing of cycle entrance is markedly crucial to avoid azoospermia due to the variations such as season, intercurrent disease and especially to fever [10, 34, 35] that can affect sperm count in the same subject. Conventional CASA semen parameter method was obviously not suitable for management of the severe oligozoospermia, particularly for Group E. Our modified procedures might be encouraged to increase opportunity to isolate more motile sperm by skipping the procedure of total sperm count. However, excess eggs were still present in several cycles in Group E due to lack of enough motile sperm and an optional solution might be through a modified hyperovulation program to reduce the number of retrieved eggs.

There has long been a concern if ICSI increases a rate of the birth defects in ICSI conceived children [36] due to potential risk of bypassing the natural selection mechanism, the transmission of mutations and current limitation of methodologies to accurately evaluate the individual sperm quality. This study had paid a close attention to evaluate sperm quality and embryonic developmental viability during the clinic and a long-term follow-up study of the birth defects of infants has initiated. We believe that development of advanced technologies such as next generation sequencing to assess the ratio of protamine 1 and protamine 2 [37] in individual sperm and analyze the genetic and epigenetic bio-information in ICSI-derived embryo can determine the individual sperm quality and select good-quality embryo for ET so that the birth defects originated from the impaired sperm may be efficiently prevented.

## Supporting Information

S1 File Original recorded information for oligozoospermia patients. The spreadsheet was representative of the translated data from original patients’ profiles that were stored in Department of Reproductive Medicine, Renji Hospital, Shanghai Jiaotong University.
(XLS)

S2 File Clinical outcomes of frozen embryo transfer. (DOC)

## References

[1] Maduro MR, Lamb DJ. Understanding new genetics of male infertility. J Urol. 2002;168(5):2197–205. . 12394759 10.1016/S0022-5347(05)64355-8

[2] Bhasin S, de Kretser DM, Baker HW. Clinical review 64: Pathophysiology and natural history of male infertility. J Clin Endocrinol Metab. 1994;79(6):1525–9. 10.1210/jcem.79.6.7989450 . 7989450

[3] Silber SJ, Nagy Z, Devroey P, Tournaye H, Van Steirteghem AC. Distribution of spermatogenesis in the testicles of azoospermic men: the presence or absence of spermatids in the testes of men with germinal failure. Hum Reprod. 1997;12(11):2422–8. . 9436677 10.1093/humrep/12.11.2422

[4] Silber SJ, Repping S. Transmission of male infertility to future generations: lessons from the Y chromosome. Hum Reprod Update. 2002;8(3):217–29. . 12078833 10.1093/humupd/8.3.217

[5] Lilford R, Jones AM, Bishop DT, Thornton J, Mueller R. Case-control study of whether subfertility in men is familial. BMJ. 1994;309(6954):570–3. 8086942 10.1136/bmj.309.6954.570 PMC2541440

[6] Ferre-Ybarz L, Basagana M, Coroleu B, Bartolome B, Cistero-Bahima A. Human seminal plasma allergy and successful pregnancy. J Investig Allergol Clin Immunol. 2006;16(5):314–6. . 17039672

[7] van der Westerlaken L, Naaktgeboren N, Verburg H, Dieben S, Helmerhorst FM. Conventional in vitro fertilization versus intracytoplasmic sperm injection in patients with borderline semen: a randomized study using sibling oocytes. Fertil Steril. 2006;85(2):395–400. 10.1016/j.fertnstert.2005.05.077 . 16595217

[8] Cao XW, Lin K, Li CY, Yuan CW. [A review of WHO Laboratory Manual for the Examination and Processing of Human Semen (5th edition)]. Zhonghua Nan Ke Xue. 2011;17(12):1059–63. . 22235670

[9] Cooper TG, Noonan E, von Eckardstein S, Auger J, Baker HW, Behre HM, et al World Health Organization reference values for human semen characteristics. Hum Reprod Update. 2010;16(3):231–45. 10.1093/humupd/dmp048. . 19934213

[10] Lu JC, Huang YF, Lu NQ. [WHO Laboratory Manual for the Examination and Processing of Human Semen: its applicability to andrology laboratories in China]. Zhonghua Nan Ke Xue. 2010;16(10):867–71. . 21243747

[11] Shu JH, Feng GX, Li J, Li JX, Gan XY, Zhang B, et al [Predictive value of sperm morphology according to WHO Laboratory Manual for the Examination and Processing of Human Semen (5th Ed) on the outcomes of IVF-ET]. Zhonghua Nan Ke Xue. 2013;19(5):414–7. . 23757962

[12] Hashimoto H, Ishikawa T, Goto S, Kokeguchi S, Fujisawa M, Shiotani M. The effects of severity of oligozoospermia on Intracytoplasmic Sperm Injection (ICSI) cycle outcome. Syst Biol Reprod Med. 2010;56(1):91–5. 10.3109/19396360903509169 . 20170289

[13] Hershko-Klement A, Rovner E, Yekutieli D, Ghetler Y, Gonen O, Cohen I, et al Embryo quality and implantation rates are not influenced by total motile count values in an ICSI programme: a novel point of view. Int J Mol Epidemiol Genet. 2012;3(3):205–12. 23050051 PMC3459219

[14] Check JH. Treatment of male infertility. Clin Exp Obstet Gynecol. 2007;34(4):201–6. . 18225678

[15] Tournaye H, Verheyen G, Nagy P, Ubaldi F, Goossens A, Silber S, et al Are there any predictive factors for successful testicular sperm recovery in azoospermic patients? Hum Reprod. 1997;12(1):80–6. . 9043908 10.1093/humrep/12.1.80

[16] Silber SJ. Microsurgical TESE and the distribution of spermatogenesis in non-obstructive azoospermia. Hum Reprod. 2000;15(11):2278–84. . 11056119 10.1093/humrep/15.11.2278

[17] Nagy ZP, Verheyen G, Tournaye H, Devroey P, Van Steirteghem AC. An improved treatment procedure for testicular biopsy specimens offers more efficient sperm recovery: case series. Fertil Steril. 1997;68(2):376–9. . 9240275 10.1016/s0015-0282(97)81534-8

[18] Zheng JF, Chen XB, Zhao LW, Gao MZ, Peng J, Qu XQ, et al ICSI treatment of severe male infertility can achieve prospective embryo quality compared with IVF of fertile donor sperm on sibling oocytes. Asian J Androl. 2015;17(5):845–9. 10.4103/1008-682X.146971 25652630 PMC4577602

[19] Pasqualotto FF, Rossi-Ferragut LM, Rocha CC, Iaconelli A Jr., Borges E Jr. Outcome of in vitro fertilization and intracytoplasmic injection of epididymal and testicular sperm obtained from patients with obstructive and nonobstructive azoospermia. J Urol. 2002;167(4):1753–6. . 11912403

[20] Pasqualotto FF, Rossi-Ferragut LM, Rocha CC, Iaconelli A Jr., Ortiz V, Borges E Jr. The efficacy of repeat percutaneous epididymal sperm aspiration procedures. J Urol. 2003;169(5):1779–81. 10.1097/01.ju.0000066849.32466.2b . 12686832

[21] Sanchez-Calabuig MJ, Lopez-Cardona AP, Fernandez-Gonzalez R, Ramos-Ibeas P, Fonseca Balvis N, Laguna-Barraza R, et al Potential Health Risks Associated to ICSI: Insights from Animal Models and Strategies for a Safe Procedure. Front Public Health. 2014;2:241 10.3389/fpubh.2014.00241 25478554 PMC4235077

[22] Devroey P, Van Steirteghem A. A review of ten years experience of ICSI. Hum Reprod Update. 2004;10(1):19–28. . 15005461 10.1093/humupd/dmh004

[23] Odom LN, Segars J. Imprinting disorders and assisted reproductive technology. Curr Opin Endocrinol Diabetes Obes. 2010;17(6):517–22. 10.1097/MED.0b013e32834040a3 20962636 PMC3124339

[24] Nagy ZP, Verheyen G, Tournaye H, Van Steirteghem AC. Special applications of intracytoplasmic sperm injection: the influence of sperm count, motility, morphology, source and sperm antibody on the outcome of ICSI. Hum Reprod. 1998;13 Suppl 1:143–54. . 9663779 10.1093/humrep/13.suppl_1.143

[25] World Health O. [Laboratory manual of the WHO for the examination of human semen and sperm-cervical mucus interaction]. Ann Ist Super Sanita. 2001;37(1):I–XII, 1–123. . 11680039

[26] Makler A. The improved ten-micrometer chamber for rapid sperm count and motility evaluation. Fertil Steril. 1980;33(3):337–8. . 6892698 10.1016/s0015-0282(16)44606-6

[27] Song SH, Chiba K, Ramasamy R, Lamb DJ. Recent advances in the genetics of testicular failure. Asian J Androl. 2016;18(3):350–5. 10.4103/1008-682X.178857 . 27048782 PMC4854078

[28] Larsen EC, Christiansen OB, Kolte AM, Macklon N. New insights into mechanisms behind miscarriage. Bmc Medicine. 2013;11 Artn 154 10.1186/1741-7015-11-154. WOS:000321200900001. PMC3699442 23803387

[29] Begueria R, Garcia D, Obradors A, Poisot F, Vassena R, Vernaeve V. Paternal age and assisted reproductive outcomes in ICSI donor oocytes: is there an effect of older fathers? Hum Reprod. 2014;29(10):2114–22. 10.1093/humrep/deu189 25073975 PMC4164148

[30] Clark DA. Is there any evidence for immunologically mediated or immunologically modifiable early pregnancy failure? Journal of Assisted Reproduction and Genetics. 2003;20(2):63–72. 10.1023/A:1021788024214. WOS:000180046800003. 12688590 PMC3455793

[31] Montagut M, Gatimel N, Bourdet-Loubere S, Daudin M, Bujan L, Mieusset R, et al Sperm freezing to address the risk of azoospermia on the day of ICSI. Hum Reprod. 2015;30(11):2486–92. 10.1093/humrep/dev234 . 26364079

[32] Vloeberghs V, Verheyen G, Haentjens P, Goossens A, Polyzos NP, Tournaye H. How successful is TESE-ICSI in couples with non-obstructive azoospermia? Hum Reprod. 2015;30(8):1790–6. 10.1093/humrep/dev139 . 26082482

[33] Negri L, Patrizio P, Albani E, Morenghi E, Benaglia R, Desgro M, et al ICSI outcome is significantly better with testicular spermatozoa in patients with necrozoospermia: a retrospective study. Gynecol Endocrinol. 2014;30(1):48–52. 10.3109/09513590.2013.848427 . 24147853

[34] Chen Z, Toth T, Godfrey-Bailey L, Mercedat N, Schiff I, Hauser R. Seasonal variation and age-related changes in human semen parameters. J Androl. 2003;24(2):226–31. . 12634309 10.1002/j.1939-4640.2003.tb02666.x

[35] Sergerie M, Mieusset R, Croute F, Daudin M, Bujan L. High risk of temporary alteration of semen parameters after recent acute febrile illness. Fertil Steril. 2007;88(4):970 e1–7. 10.1016/j.fertnstert.2006.12.045 . 17434502

[36] Basille C, Frydman R, El Aly A, Hesters L, Fanchin R, Tachdjian G, et al Preimplantation genetic diagnosis: state of the art. Eur J Obstet Gynecol Reprod Biol. 2009;145(1):9–13. 10.1016/j.ejogrb.2009.04.004 . 19411132

[37] Rogenhofer N, Dansranjavin T, Schorsch M, Spiess A, Wang H, von Schonfeldt V, et al The sperm protamine mRNA ratio as a clinical parameter to estimate the fertilizing potential of men taking part in an ART programme. Hum Reprod. 2013;28(4):969–78. 10.1093/humrep/des471 . 23340056

