# Correction: Decreased Sperm Motility Retarded ICSI Fertilization Rate in Severe Oligozoospermia but Good-Quality Embryo Transfer Had Achieved the Prospective Clinical Outcomes

**DOI:** 10.1371/journal.pone.0165684

**Published:** 2016-10-25

**Authors:** Jufen Zheng, Yongning Lu, Xianqin Qu, Peng Wang, Leiwen Zhao, Minzhi Gao, Huijuan Shi, Xingliang Jin

The first author's name and fifth author's name are spelled incorrectly. The correct name of the first author is Jufen Zheng. The correct name of the fifth author is Leiwen Zhao. The correct citation is: Zheng J, Lu Y, Qu X, Wang P, Zhao L, Gao M, et al. (2016) Decreased Sperm Motility Retarded ICSI Fertilization Rate in Severe Oligozoospermia but Good-Quality Embryo Transfer Had Achieved the Prospective Clinical Outcomes. PLoS ONE 11(9): e0163524. doi:10.1371/journal.pone.0163524
