# Supplementary material for: Decreased Sperm Motility Retarded ICSI Fertilization Rate in Severe Oligozoospermia but Good-Quality Embryo Transfer Had Achieved the Prospective Clinical Outcomes
Source: PLoS One. 2016 Sep 23;11(9):e0163524. doi: 10.1371/journal.pone.0163524 (PMC5035010; doi:10.1371/journal.pone.0163524)
Supplement: S2 File — (DOC) [file pone.0163524.s002.doc]

**S2 File: Clinical outcomes of frozen embryo transfer**

| **Group** | **Frozen**  **Method** | **Thawed Embryos** | | **Recovery Embryos** | | **ET** | | **Pregnancy** | | **Live Birth** | |
| --- | --- | --- | --- | --- | --- | --- | --- | --- | --- | --- | --- |
|  |  | **Cycle** | **N** | **N** | **Rate** | **Cycle** | **Mean** | **Mean** | **N** | **Mean** | **N** |
| **A** | PC | 48 | 133 | 112 | .8421* | 46 | 2.35 | .3043 | 14 | .2609 | 12 |
|  | VC | 23 | 60 | 58 | .9667 | 22 | 2.41 | .4091 | 9 | .3182 | 7 |
|  | Total | 71 | 193 | 170 | .8808 | 68 | 2.37 | .3382 | 23 | .2794 | 19 |
| **B** | PC | 24 | 70 | 61 | .8714* | 23 | 2.35 | .3043 | 7 | .2174 | 5 |
|  | VC | 18 | 39 | 35 | .8974 | 17 | 2.05 | .3529 | 6 | .3529 | 6 |
|  | Total | 42 | 109 | 96 | .8807 | 40 | 2.23 | .3250 | 13 | .2750 | 11 |
| **C** | PC | 126 | 386 | 301 | .7798* | 116 | 2.47 | .3362 | 39 | .3017 | 35 |
|  | VC | 63 | 163 | 143 | .8773 | 62 | 2.26 | .3548 | 22 | .2742 | 17 |
|  | Total | 189 | 549 | 444 | .8087 | 178 | 2.39 | .3427 | 61 | .2921 | 52 |
| **D** | PC | 47 | 153 | 114 | .7451* | 46 | 2.48 | .3478 | 16 | .2609 | 12 |
|  | VC | 43 | 112 | 107 | .9554 | 41 | 2.32 | .4634 | 19 | .3659 | 15 |
|  | Total | 90 | 265 | 221 | .8340 | 87 | 2.40 | .4023 | 35 | .3103 | 27 |
| **E** | PC | 26 | 75 | 59 | .7867* | 25 | 2.28 | .2000 | 5 | .1600 | 4 |
|  | VC | 22 | 56 | 54 | .9643 | 22 | 2.45 | .3636 | 8 | .2727 | 6 |
|  | Total | 48 | 131 | 113 | .8626 | 47 | 2.36 | .2766 | 13 | .2128 | 10 |
| **Total** | PC | 271 | 817 | 647 | .7919* | 256 | 2.42 | .3164 | 81 | .2656 | 68 |
|  | VC | 169 | 430 | 397 | .9233 | 164 | 2.30 | .3902 | 64 | .3110 | 51 |
|  | Total | 440 | 1247 | 1044 | .8359 | 420 | 2.37 | .3452 | 145 | .2833 | 119 |

The table was representative of the clinical outcomes of FET before the end of 2011 with two different embryo freezing methods: slow programming cryopreservation (PC) and vitrification cryopreservation (VC). PC was mainly performed before 2009. Recovery rate of frozen embryo was comparable lower with PC method than VC (* p < 0.001), which was not affected by groups. The rates of pregnancy and live-birth were lower in PC than VC, although they were not statistical.
